# Qualitative and Quantitative Anatomical Analysis of the Constitutive Bark of *Q. ilex* x *Q. suber* Hybrids

**DOI:** 10.3390/plants11192475

**Published:** 2022-09-22

**Authors:** Gonzalo de Burgos, Eduardo Díez-Morales, Unai López de Heredia, Álvaro Soto

**Affiliations:** Dpto. Sistemas y Recursos Naturales, ETSI Montes, Forestal y del Medio Natural, Universidad Politécnica de Madrid, Ciudad Universitaria s/n, 28040 Madrid, Spain

**Keywords:** cork, hybridization, *Quercus*, rhytidome, suberization

## Abstract

Hybridization and introgression between cork oak (*Quercus suber*) and holm oak (*Q. ilex*) have traditionally been reckoned as undesirable processes, since hybrid individuals lack the profitable bark characteristics of cork oak. Nevertheless, a systematic and quantitative description of the bark of these hybrids at the microscopic level, based on a significant number of individuals, is not available to date. In this work we provide such a qualitative and quantitative description, identifying the most relevant variables for their classification. Hybrids show certain features intermediate between those of the parent species (such as phellem percentage in the outer bark, which was approximately 40% as a mean value for hybrids, 20% in holm oak and almost 99% in cork oak), as well as other unique features, such as the general suberization of inactive phloem (up to 25% in certain individuals), reported here for the first time. These results suggest a relevant hybridization-induced modification of the genetic expression patterns. Therefore, hybrid individuals provide a valuable material to disentangle the molecular mechanisms underpinning bark development in angiosperms.

## 1. Introduction

Cork oak (*Quercus suber* L.) and holm oak (*Q. ilex* L.) are two ecologically, economically, environmentally and socially relevant oak species that are key elements in open woodlands from the western Mediterranean basin. Both species differ in morphological traits of bark, cupules of the acorns, or leaves [1], and they belong to two different subsections (Ilex and Cerris) within the Cerris section [2,3].

Possibly, bark anatomy is the most discriminating phenotypic character between cork and holm oaks at the adult stage. The bark of most Euromediterranean oak species is made up of a rhytidome, a structure formed by successive phellogens (likely induced by the mechanical tensions derived from the activity of vascular cambium), producing thin, suberized and intricate phellem layers enclosing heterogeneous cortical tissues (parenchyma, fibers, etc.) and collapsed phloem cells. This type of outer bark has been described in detail for *Q. robur* [4], *Q. petraea* [5], *Q. faginea* [6], *Q. cerris* [7] and *Q. ilex* ssp. *rotundifolia* [8].

In contrast, the outer bark of *Q. suber* is thought to be caused by the differentiation of a single long-living phellogen that generates new layers of suberized and sometimes lignified cells each year, rather than producing a rhytidome [9]. This bark differentiation pattern results in cork, a thick regular suberized material with extraordinary properties of protection and insulation against pathogen attacks or fire [10,11]. Cork is mainly formed by dead, empty prismatic cells that are stacked by their bases in radially aligned rows disposed in parallel without intercellular spaces [12]. The cork cell walls are covered with suberin, an inert hydrophobic substance partially similar to lignin and cutin. Virgin cork is more irregular and presents more lignified cells than the traumatic cork that is produced after successive peeling, which is the basis of commercial cork exploitation. Indeed, cork is exploited in the Mediterranean countries due to its insulating properties and renewable character, reaching high economic and social values in rural areas [10].

Although *Q. suber* and *Q. ilex* have slightly different ecological constraints, they frequently co-exist, forming mixed stands (particularly with ssp. *rotundifolia*) where they are able to outcross to produce fertile hybrids [13]. The occurrence of *Q. ilex* x *suber* hybrids (*Quercus x morisii* Borzí; *Quercus* x *avellaniformis* Colmeiro & E. Boutelou) has long been known and botanical descriptions present these hybrids as having viable acorns with conical cupules with free bracts, glabrescent leaves of light green tone and strongly cracked bark [14,15,16,17,18]. These hybrids also present micro-morphological and anatomical characters related to the presence/absence and distribution of foliar trichomes [19] and to the thickness of the leaf lamina [20].

Traditionally, it has been considered that hybridization produces poor quality cork and that first-generation hybrids are not suitable for cork extraction [21]. Early qualitative studies of the bark anatomy of *Q. ilex* x *suber* hybrids reported high variability and described some hybrids showing corky barks, others resembling the rhytidome structure of *Q. ilex*, but with thicker layers of phellem, and some others presenting intermediate traits [17]. However, a robust quantitative description of the anatomical features of the bark of hybrids is lacking. To our knowledge, this issue has only been approached from a qualitative perspective, using a limited number of putative hybrids [17,22], the precise level of hybridization of which could not be checked with molecular markers [23].

In the present paper, we adopt a qualitative and quantitative approach to assess bark anatomical features of a higher number of adult *Q. ilex* x *suber* hybrids (20 individuals) of known hybridization level inferred with high resolution molecular markers [24].

## 2. Results and Discussion

### 2.1. Preliminary Observations

**Figure 1:**
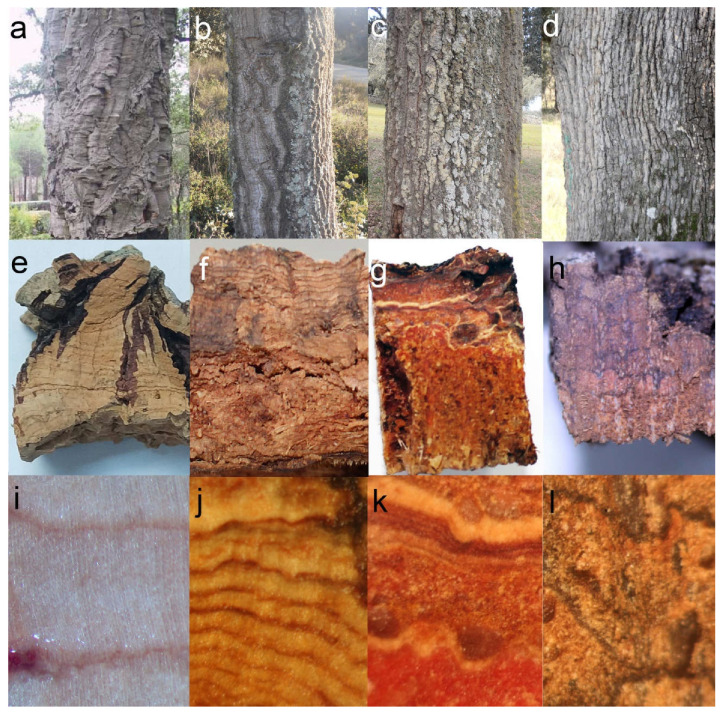
Barks of *Q. suber* (**a**,**e**,**i**), suber-like hybrid (**b**,**f**,**j**), ilex-like hybrid (**c**,**g**,**k**) and *Q. ilex* ssp. *rotundifolia* (**d**,**h**,**l**) as seen in the tree (**a**–**d**), and in transverse section, both *de visu* (**e**–**h**) and under magnifying glass (**i**–**l**).

Our observations of *Q. suber* samples were consistent with the descriptions reported in previous works [10,17]. The outer bark of this species is formed by a single periderm, several centimeters thick, and with deep cracks and furrows, mainly longitudinal, not reaching the phellogen. This periderm appears, in a transverse section, as a light-colored layer, where annual growth rings can be distinguished as darker lines. Microscopically, the periderm appears to be formed mainly of empty, suberized cells, with few schlerenchymatic nodules (Figure 1a,e,i).

On the contrary, *Q. ilex*, as with most species of the genus, shows a rhytidome, with several successive anastomosed periderms and lignified inactive phloem in between. Macroscopically, holm oak outer bark is finely crackled in small tiles, which eventually fall from the tree. In a transverse section, periderms can be distinguished as dark, thin lines; inactive phloem presents a brown-reddish color, with whitish multiseriate lignified rays and schlerenchymatic nodules, consistent with previous observations [8]. As seen under the microscope, periderms are a few cells thick, and become more abundant in the outer part of the bark, as mechanical tensions induce the differentiation of new phellogens in the inactive phloem between periderms (Figure 1d,h,l).

**Figure 2:**
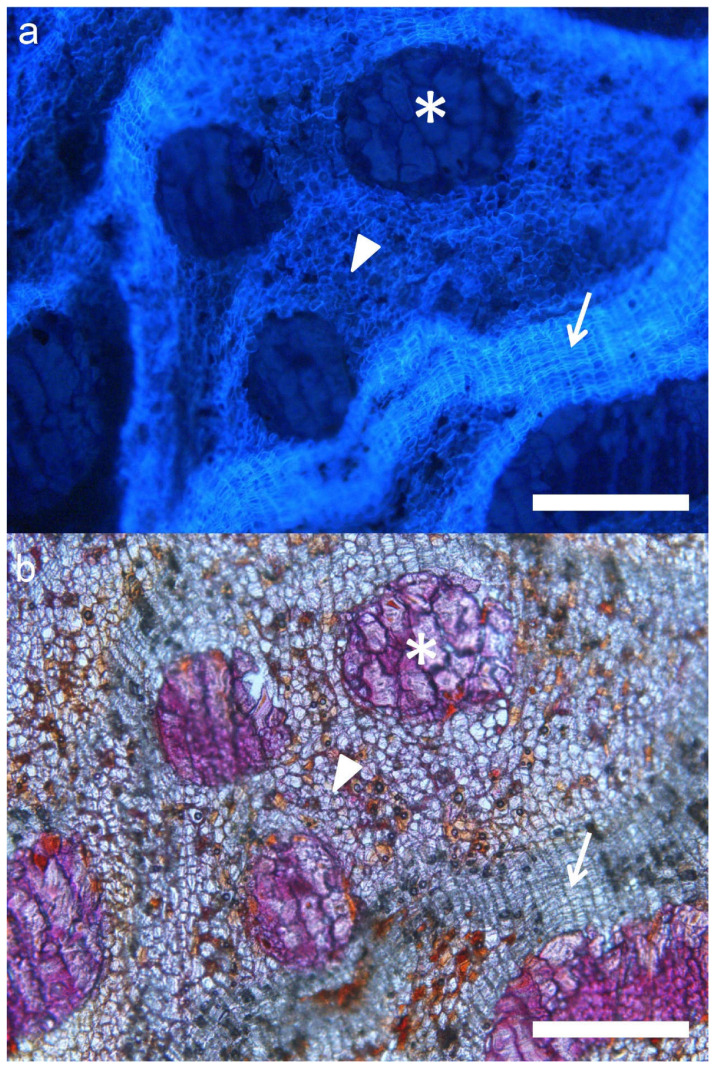
Cross section of the bark of a hybrid tree under UV (**a**) and visible light (**b**). Periderms (arrow), schlerenchymatic nodules (asterisk), and a high proportion of suberized inactive phloem (arrowhead) can be seen. Bar scale: 200 μm.

Much larger variability can be observed in hybrid individuals. Externally, most of them show an intermediate aspect, with deeper cracks than *Q. ilex* bark, and larger tiles. Some individuals present longitudinal furrows more similar to those of *Q. suber*, and with an orange color in the inner part. In transverse sections, most individuals show abundant light-colored layers, corresponding to periderms. Very often they appear arranged in parallel, although in other cases they look anastomosed. Abundant whitish multiseriate rays and schlerenchymatic nodules are also visible within inactive phloem (Figure 1b,c,f,g,j,k). Under the microscope, most individuals show very abundant periderms, often much thicker than in *Q. ilex*. These periderms also appear much closer one to another than in holm oak. More strikingly, most individuals also present suberization in inactive phloem cells (Figure 2). This feature has not been observed in *Q. suber* or *Q. ilex* samples, or in any other species, to our knowledge.

### 2.2. Systematic Measurements and Analysis

**Figure 3:**
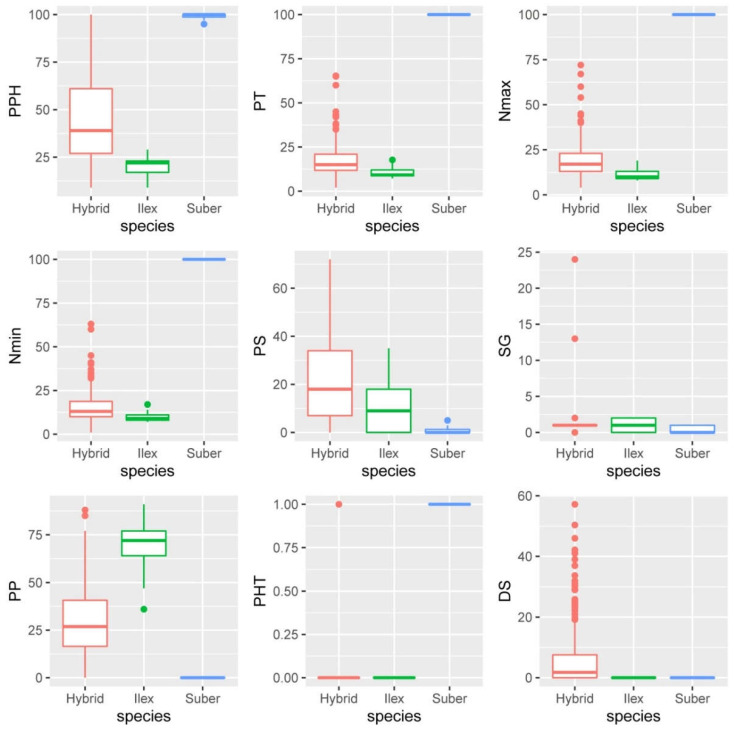
Boxplots of *Q. ilex*, *Q. suber* and the hybrids for all the variables related to outer bark anatomy described in Table 2.

Once explorative observations had been performed, we defined a series of qualitative and, mostly, quantitative parameters or variables in order to perform a statistical comparison of the barks. Measurements of these variables showed noticeable differences between the samples of the two parental species, as well as large variability among the hybrid trees (Figure 3; Table S1). Actually, within individual variability was also detected in hybrid trees, with some individuals showing areas with thicker phellems and other areas with higher presence of inactive phloem and thinner periderms. Values observed for parental species were consistent with observations reported previously [8,9,12,13]. Thus, *Q. suber* samples were characterized by a very thick periderm, accounting for most of the outer bark, with a large, indefinite number of phellem cells (set to 100 in order to ease further numerical analysis), with a low proportion of schlerenchymatic cells. A larger proportion of non-suberized inactive phloem (almost 70% of their outer bark) characterized *Q. ilex* samples, with a significant proportion of lignified rays and schlerenchymatic nodules (9–14%). Phellem tissue accounted for approximately 20% of the bark transverse area and was comprised of several thin (~10 cells thick), anastomosed periderms, which was consistent with previous descriptions [8], and similar to other *Quercus* species, as, for instance, *Q. petraea* [5].

ANOVA analyses showed significant differences for all the quantitative variables regarding species and individuals (Table 1), and so did the Kruskal-Wallis test for the qualitative variables.

**Table 1:** Summary of the ANOVA results for the model expressed in Equation (1) and the quantitative variables described in Table 2. df: degrees of freedom; SS: sum of squares; MS: mean square; F: value of the F-test.

Variable	Factor	df	SS	MS	F	Pr (>F)
**PPH**	α_i_ (species)	2	46,228	23,114	8.4	0.002
	β_j(i)_ (species:ind)	20	89,269	4463	16.9	0.001
	γ_ijk_ (residuals)	368	97,207	264		
**PT**	α_i_ (species)	2	81,547	40,774	144.0	0.001
	β_j(i)_ (species:ind)	20	8798	440	7.9	0.001
	γ_ijk_ (residuals)	368	20,525	56		
**Nmax**	α_i_ (species)	2	78,133	39,066	129.6	0.001
	β_j(i)_ (species:ind)	20	9342	467	7.6	0.001
	γ_ijk_ (residuals)	368	22,523	61		
**Nmin**	α_i_ (species)	2	84,678	42,339	155.6	0.001
	β_j(i)_ (species:ind)	20	8458	423	8.0	0.001
	γ_ijk_ (residuals)	368	19,549	53		
**PP**	α_i_ (species)	2	36,622	18,311	17.3	0.001
	β_j(i)_ (species:ind)	20	32,934	1647	8.1	0.001
	γ_ijk_ (residuals)	368	74,518	202		
**PS**	α_i_ (species)	2	5365	2682	2.3	0.119
	β_j(i)_ (species:ind)	20	35,762	1788	8.3	0.001
	γ_ijk_ (residuals)	368	78,966	215		
**DS**	α_i_ (species)	2	948	473.8	1.5	0.242
	β_j(i)_ (species:ind)	20	9757	487.8	7.9	0.001
	γ_ijk_ (residuals)	368	22,753	61.8		

**Table 2:** Description of the measured traits for outer bark anatomical characterization. VL: visible light; UV: Ultra-violet light.

Trait	Description	Microscope Light Exposure
PPH	Percentage of ordered suberized cells (phellem cells)	UV
PT	Mean phellem cell number	UV
Nmax	Maximum number of phellem cells	UV
Nmin	Minimum number of phellem cells	UV
PHT	1: Rhytidome with non-functional phloem 2: Rhytidome with massive phellem	Both
PP	Percentage of parenchymatic cells	Both
PS	Percentage of schlerenchymatic cells	VL
SG	0: lack of schlerenchymatic cells 1: isolated schlerenchymatic cells 2: grouped schlerenchymatic cells	VL
DS	Percentage of disordered suberized tissue	UV

**Figure 4:**
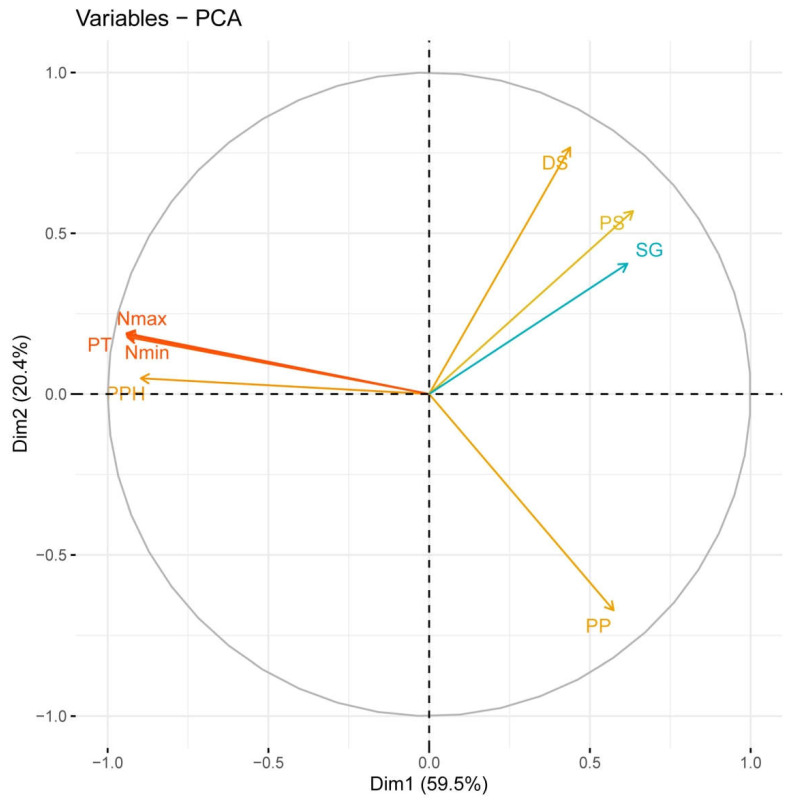
Relative contribution of the original variables to the first two PCs for the multivariate analysis. The percentage of variance explained by each principal component is indicated.

Relationships among the variables were assessed by means of a correlation analysis (Table S2) Thus, phellem average thickness was highly correlated with maximum and minimum number of phellem cells and with the qualitative variable “phellogen type” (PHT), as expected, and negatively with the proportion of other tissues. This was consistent with Principal Component Analysis. The first two PCs explained 80% of the total variance; the first one (59.5% of total variance) was mainly composed of variables related to phellem characteristics: phellem average thickness, maximum and minimum number of phellem cells and percentage of phellem tissue. Variables related to the other cell types contributed mainly to the second component (20.4%): percentages of parenchyma and, oppositely, percentages of schlerenchyma and suberized inactive phloem (although correlation of this suberization with the percentage of other tissues was not statistically significant) (Figure 4).

**Figure 5:**
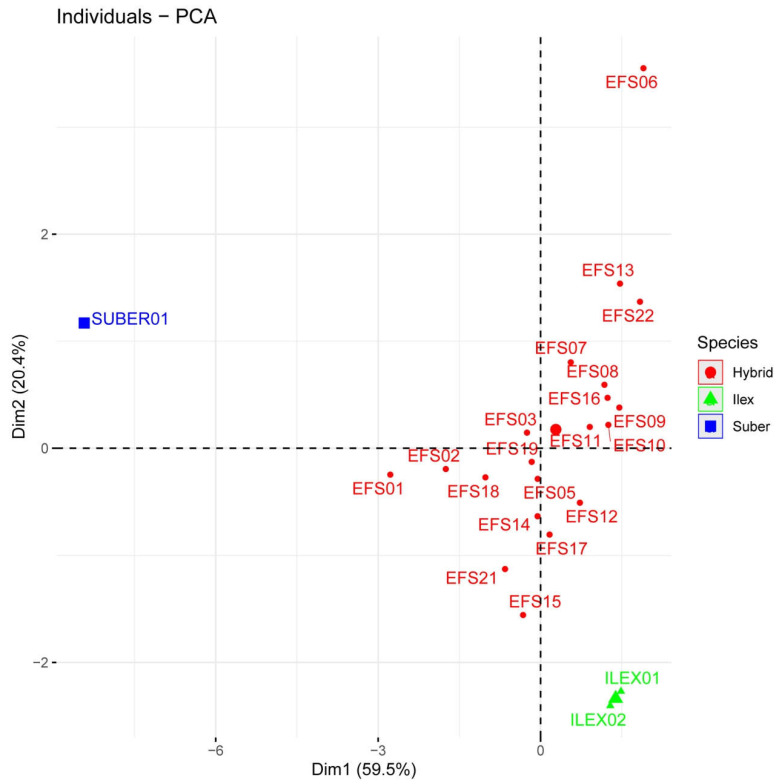
Biplot of the mean individual scores for the first two PCs for the multivariate analysis. The percentage of variance explained by each principal component is indicated.

The first component clearly discriminated *Q. suber* from *Q. ilex* and most hybrid individuals (Figure 5). However, certain hybrids, such as FS01 and, to a minor extent, FS02 and FS18, were also separated from the rest of hybrids with this component. Notwithstanding, the average phellem thickness and maximum and minimum number of phellem cells in *Q. suber* were much higher than in the hybrids (and, therefore, cork oak samples appeared far away in that axis). FS01, FS02 and FS18 had a much higher proportion of phellem in their barks than the other hybrids. This was especially the case with FS01, which presented almost 92% of the bark as being occupied by suberized, ordered cells (“percentage of phellem”, PPH). This tissue also represented 67 and 64% of the bark in FS02 and FS18 samples, respectively, much larger than in the other hybrids. Average phellem thickness in these three hybrids was not that high, but in FS01 and FS02 (>28 cells) it was 1.5–2.5 times thicker than in most other individuals. On the contrary, FS01 showed a very low proportion of other cell types in its bark, and almost no suberization of the remaining inactive phloem, as evident in FS18.

**Figure 6:**
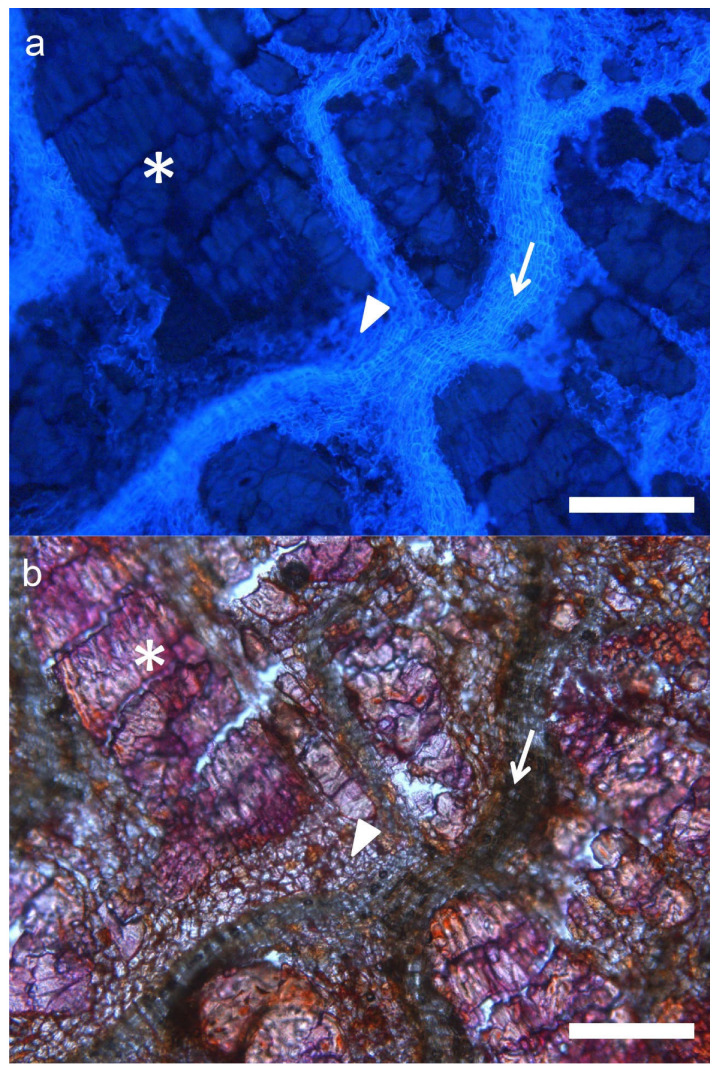
Cross section of the bark of a hybrid tree under UV (**a**) and visible light (**b**). Periderms (arrow), a large proportion of schlerenchymatic nodules (asterisk), and a lower proportion of suberized inactive phloem (arrowhead) can be appreciated. Bar: 100 μm.

The second component allowed the discrimination of *Q. ilex* and hybrids and, among the latter, distinguishing of the hybrids with this unique phloem suberization from those more similar to *Q. ilex*. Thus, FS06 and FS22 showed a large proportion of these cells (25% and 14% of their barks, respectively) (Figure 2). Interestingly, these individuals showed the lowest proportion of phellem in their barks, and the thinnest periderms, close to *Q. ilex* values. The other variable contributing in the same sense to this component was the proportion of schlerenchymatic tissues in the bark, which reached up to 42% in FS13 (also with >30% of phellem), and values above 30% in FS06, FS08, FS16 and FS22 (Figure 6).

**Figure 7:**
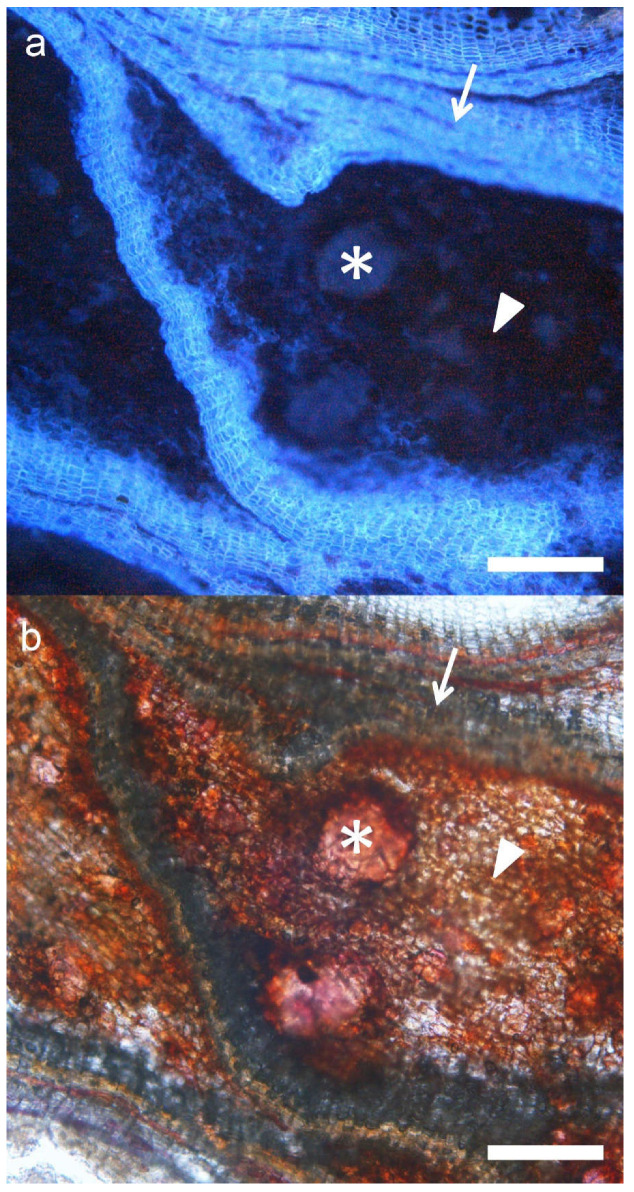
Cross section of the bark of a hybrid tree under UV (**a**) and visible light (**b**). Periderms (arrow), schlerenchymatic nodules (asterisk), and non-suberized inactive phloem (arrowhead) can be appreciated. Bar: 100 μm.

On the contrary, the other individuals showed slightly thicker periderms (30–50% of bark), and the largest proportions (more than 40% in some trees, and always >30%) of non-suberized parenchymatic tissue (inactive phellem), in their rhytidomes. This was the case, for instance, with FS15, FS17 and FS21, where inactive phloem suberization was very low (2.83, 1.15 and 0.12% of the bark) (Figure 7).

**Figure 8:**
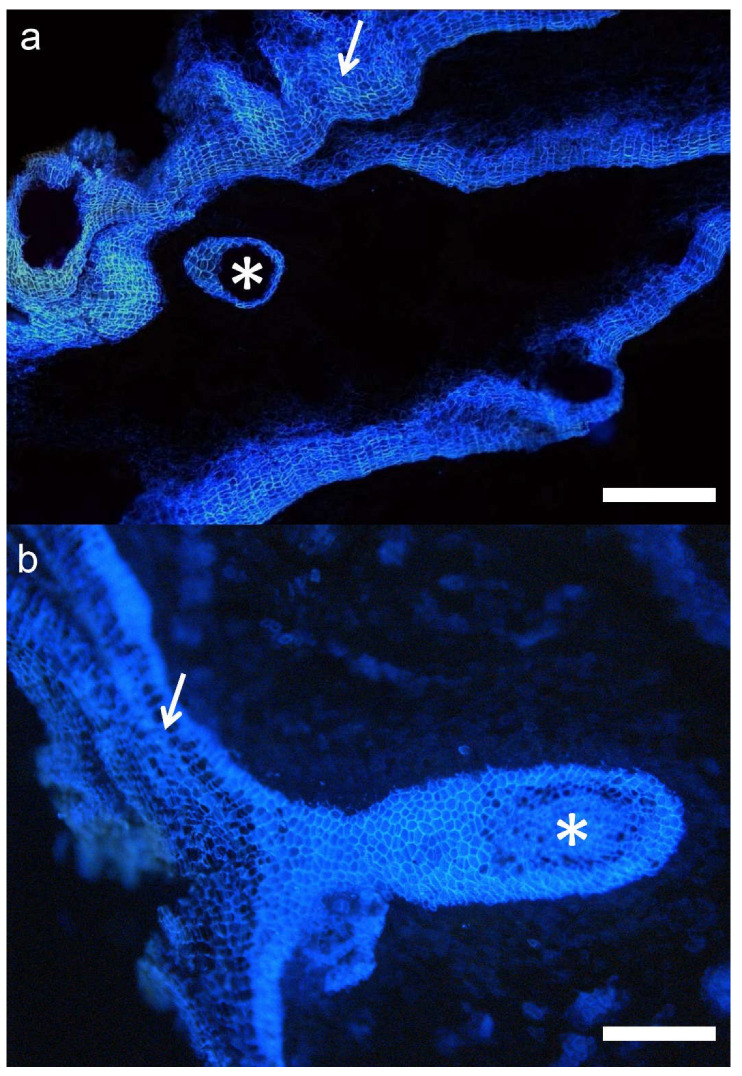
Cross section of the bark of a hybrid tree under UV light. (**a**): A closed thin periderm surrounding a schlerenchymatic nodule (asterisk) can be appreciated. Bar: 100 μm (**b**): A similar structure, connected to the normal periderm (arrow). Bar: 50 μm.

Other remarkable structures were observed in the hybrids. For instance, schlerenchymatic nodules were often surrounded by thin periderms, and could be observed both in transverse and radial sections (Figure 8). These formations appeared preferentially in the outermost part of the bark, and could differentiate as an induced defense against a mechanical injury. The damaged area would be isolated by a traumatic periderm and then lignified, as described for conifers [25,26,27]; alternatively, they could be formed constitutively. These observations were consistent with the “independent periderms” mentioned in previous work [17].

The relationship between these bark anatomical variables and the contribution of each of the parental species to the genome of hybrid trees was also investigated. This contribution was estimated previously [24], based on 9251 nuclear markers, mostly SNP. Nevertheless, only slight correlations were obtained (with R^2^ values below 20%) and were mostly due to the presence of FS01. This was the only individual classified as a probable backcross with *Q. suber*, with a 65–73.1% contribution of this species to its genome (qs). Consistently, this individual was characterized by a very large proportion of phellem in its bark (>90%) and an extremely low proportion of schlerenchyma (3%). Additionally, almost no suberization of the inactive phloem was detected (0.6% of the bark). If this individual was removed from the analysis, no significant relationship was detected for any of the variables. The other individuals were presumably first-generation hybrids, with estimated *Q. suber* genomic contributions (qs) between 45.4 and 52.6%. Lack of correlation was not surprising, due, firstly, to the narrow range of qs, and, secondly, to its global character. This parameter provided an estimation of the average contribution of *Q. suber* to the whole genome, notwithstanding higher or lower contributions to specific genomic regions (not expected for F1 hybrids, though) and, more importantly, to the transcriptome, and consequently, to the proteome or metabolome.

### 2.3. Concluding Remarks

Development of a thick and corky bark, such as the one shown by *Q. suber*¸ relies on the presence of an active, long-living phellogen, able to produce enough anticlinal divisions in order to increase its circumference and keep the pace with the growth rhythm imposed by the activity of vascular cambium. Thus, the tangential tensions endured by each phellogen cell, inducing the differentiation of new successive inner phellogens in other species, such as *Q. ilex*, would remain moderate in *Q. suber*¸ and the periclinal divisions of its single phellogen, accumulated over the years, would give rise to the characteristic thick phellem of this species.

All the hybrid trees analysed here, excepting FS01, were presumably first-generation hybrids, according to previous work [24]. Therefore, they are expected to carry a copy of all the genes underpinning the formation of a thick corky bark, as in *Q. suber*. Combination with *Q. ilex* genes hampers, however, the development of such a bark in these trees. Maybe trees need to have two copies of certain genes or alleles in order to produce corky bark. Indeed, the bark of FS01, the only individual classified as a backcross with *Q. suber* (and expected, therefore, to carry two *Q. suber* alleles in 50% of its loci, on average) is far more similar to cork oak bark. In addition, hybridization itself can induce epigenetic modifications, as pointed out previously in [24], which could affect the expression of such genes.

Thus, among F1 hybrids we also detected relevant differences in their barks, regarding the number and thickness of their periderms, or the proportion of parenchymatic and sclerenchymatic cells in the rhytidome. Probably, the most striking feature was the generalized suberization of inactive phloem, shown by several individuals. This feature also points to the modification of the regulation pathways of suberin incorporation to the cell wall, induced by hybridization. Hybrid individuals appear, therefore, as an extremely interesting material for further research on the regulation of bark development, mainly by comparison with the parental species, *Q. suber* and *Q. ilex*.

## 3. Materials and Methods

### 3.1. Sampling and Laboratory Procedures

Twenty naturally grown hybrid trees were identified and sampled in a mixed holm oak–cork oak forest in Fregenal de la Sierra (Extremadura, SW Spain). Hybrids were initially identified according to morphological characters described previously [17], and further confirmed with molecular markers [24]. Samples were also collected from one cork oak (*Q. suber*) and two holm oak (*Q. ilex* ssp. *rotundifolia*) trees as a reference, since previous works reported almost no variability among individuals within these species [8]. Outer bark samples (5 cm × 12 cm) were harvested from the south-facing part of the trees at DBH (1.30 m) using a hammer and a chisel.

Six cubical prisms (2 cm × 4 cm) were extracted from each sampled bark piece. After polishing, the cube surface samples were submerged in 70% ethanol and surface photographs were taken with a Moticam 1080 camera on a Nikon 8MZ-2T binocular lens. In addition, 25 μm thick cross-sections were obtained with a Leica SM2400 microtome. Cross-sections were heated in a sodium hypochlorite solution prior to staining for suberized and lignified tissue observation, using an Olympus BX51 microscope (Olympus Corporation, Tokyo, Japan) equipped with 4×, 10×, 40×, 60×, 100× objectives and 10× ocular, and with a fluorescence lamp Olympus U-RFL-T (Olympus Corporation, Tokyo, Japan) (excitation at 340–380 nm and 410–450 nm barrier filters) and a Moticam 1080 camera (Motic Microscopy, Xiamen City, China). The phloroglucinol-HCl test [28] was performed for visualization of lignified and suberized cells under tungsten and UV light. A drop of 1% phloroglucinol:ethanol solution (*w*/*v*) was poured on washed crossed-sections, followed by the addition of 25 μL of 35% HCl. Using this procedure lignin appeared stained in red, while quenching of lignin autofluorescence under UV light by phloroglucinol-HCl allowed the identification of suberized tissue [29].

### 3.2. Quantitative Analysis

The obtained photographs were treated with the Motic Images Plus 2.0 (Motic Microscopy, Xiamen City, China) and ImageJ [30] software applications to measure seven bark quantitative and two qualitative categorical traits (Table 2), based on a preliminary exploratory analysis of the most relevant traits for outer bark characterization in cork oak, holm oak and their hybrids [13]. For each sample, measurements were performed in four random intact points. In total, 407 pairs of photographs, obtained with visible light and ultraviolet light, were acquired.

A categorical variable (PHT) evaluated de visu the type of bark in the sample (1: rhytidome with non-functional phloem; 2: rhytidome with massive phellem cells). Phellem related quantitative traits scored the percentage of phellem cells in the sample (relative to the area of the section) (PPH), the mean thickness of phellem tissue from measures in four random points of the photograph (PT) and the maximum and minimum number of phellem cells (Nmax and Nmin) in each sample. Cork oak presented a massive phellem that occupied the entire photograph; therefore, an arbitrary PT = 100 cells was established to allow the comparison with the phellem thickness of the rhytidomes of holm oak and the hybrids. The percentage of non-functional phloem tissue between successive periderms (PP) was estimated using both visible and UV lights. Photographs taken under visible light were also employed to ascertain the percentage of lignified schlerenchymatic cells (PS) and their spatial arrangement through a categorical variable (SG), as they may occur isolated (1), forming groups of lignified cells (2), or not occur at all (0). Finally, providing that some of the hybrid samples showed disordered suberized tissue, we calculated the percentage of this type of cells in the sample (DS).

Median, mean, maximum/minimum values and standard deviation were scored for each quantitative variable at individual tree and species (cork oak, holm oak and hybrids) levels, respectively. To identify the most significant variables discriminating between bark types, ANOVAs were performed for each quantitative variable, according to the following model:

(1) $$Y_{ijk}=\mu +\alpha_{i}+\beta_{j\left(i\right)}+\gamma_{ijk},$$

where *μ* is the mean value of the variable *Y*, *αi* is the effect of species *i*, *βj(i)* is the effect of the individual *j* within species *i* and *γijk* is the error term for the observation *k* of the individual *j* within species *i*. The differences between species and individuals for the categorical variables PHT and SG were evaluated using a Kruskal-Wallis test.

A principal component analysis (PCA) was performed by scaling the original variables with the “prcomp” function from the R 4.0.3 “stats” package (R Core Team 2020). The two principal components (PCs) explaining most of the total variance were selected. New variables were created from the scores of the components, thus simplifying further analysis. The mean scores by individual of the first two principal components were bi-plotted to graphically inspect differences between species, and the correlation of these values with the introgression level of the hybrid trees estimated by López de Heredia et al. (2020). The introgression level was estimated using the qs coefficient provided by a Bayesian approach implemented in STRUCTURE v. 2.3.4 [31] for each individual that showed values between 0 and 1. Qs values close to 0 suggested ilex-like trees, qs values close to 1 suggested suber-like individuals and qs values close to 0.5 suggested the tree was a first generation (F1) hybrid.

## Data Availability

Not applicable.

## Supplementary Materials

The following supporting information can be downloaded at: https://www.mdpi.com/article/10.3390/plants11192475/s1, Table S1: Mean and standard deviation values (among brackets) for the anatomical variables of the barks described in Table 1; Table S2: Pearson correlation estimate between the anatomical variables of the barks described in Table 1.
