# Supplementary material for: Qualitative and Quantitative Anatomical Analysis of the Constitutive Bark of Q. ilex x Q. suber Hybrids
Source: Plants (Basel). 2022 Sep 22;11(19):2475. doi: 10.3390/plants11192475 (PMC9572208; doi:10.3390/plants11192475)
Supplement: Supplementary file 1 [file plants-11-02475-s001.zip › plants-1931364-supplementary.pdf]

Supplemental material

Table S1: Mean and standard deviation values (among brackets) for the anatomical variables of the barks described in Table 1.

species	ind	PPH	PHT	PT	Nmax	Nmin	DS	PS	SG	PP
Hyb	EFS01	91,67 (7,78)	0,27 (0,46)	28,49 (11,38)	29,20 (11,05)	27,73 (11,73)	0,60 (1,3)	3,00 (2,88)	0,73 (0,46)	4,73 (7,06)
Hyb	EFS02	67,04 (17,83)	0 (0)	28,35 (15,48)	31,42 (16,54)	25,83 (14,61)	2,74 (7,57)	10,54 (11,94)	0,75 (0,61)	19,68 (11,83)
Hyb	EFS03	59,25 (15,34)	0 (0)	16,25 (2,70)	18,25 (3,35)	14,60 (2,93)	4,33 (5,54)	15,60 (11,27)	1,15 (0,59)	20,82 (8,35)
Hyb	EFS05	40,08 (15,56)	0 (0)	21,11 (6,82)	23,92 (7,76)	18,08 (6,95)	7,15 (9,91)	14,75 (15,90)	0,83 (0,72)	38,02 (19,02)
Hyb	EFS06	27,75 (13,57)	0 (0)	15,15 (5,00)	16,69 (5,02)	13,63 (4,76)	24,95 (14,30)	32,38 (12,07)	1,63 (0,50)	14,93 (14,48)
Hyb	EFS07	42,32 (18,37)	0 (0)	16,80 (6,45)	18,23 (6,33)	15,27 (6,43)	8,68 (13,06)	22,95 (17,76)	1,23 (0,61)	26,05 (15,04)
Hyb	EFS08	36,25 (14,81)	0 (0)	11,03 (4,51)	12,58 (4,50)	9,58 (4,93)	5,51 (5,08)	31,17 (14,86)	1,17 (0,72)	27,07 (14,31)
Hyb	EFS09	36,26 (15,22)	0 (0)	13,32 (4,84)	15,35 (6,07)	11,39 (3,66)	5,81 (6,45)	18,17 (13,17)	2,09 (4,82)	39,75 (12,63)
Hyb	EFS10	40,18 (17,9)	0 (0)	12,75 (4,93)	14,73 (4,90)	11,18 (5,04)	7,07 (11,15)	12,91 (10,58)	2,00 (3,69)	39,84 (11,54)
Hyb	EFS11	30,89 (14,88)	0 (0)	16,18 (6,95)	18,72 (7,58)	14,22 (7,11)	5,16 (4,83)	27,83 (15,49)	1,11 (0,32)	36,12 (12,91)
Hyb	EFS12	32,47 (14,46)	0 (0)	13,48 (4,66)	15,80 (4,81)	11,73 (4,82)	7,16 (8,75)	18,47 (18,79)	0,73 (0,59)	41,90 (14,07)
Hyb	EFS13	29,00 (15,36)	0 (0)	13,78 (8,50)	15,68 (8,74)	12,04 (8,28)	7,10 (6,56)	42,36 (17,4)	1,25 (0,52)	21,55 (11,05)
Hyb	EFS14	44,70 (27,34)	0 (0)	16,02 (6,42)	17,95 (7,11)	14,55 (6,21)	5,98 (13,40)	14,00 (18,68)	0,60 (0,60)	35,32 (20,74)
Hyb	EFS15	43,90 (18,45)	0 (0)	17,52 (8,35)	19,20 (8,57)	16,00 (8,21)	2,83 (5,84)	8,50 (13,75)	0,5 (0,71)	44,77 (20,23)
Hyb	EFS16	32,10 (13,79)	0 (0)	12,23 (3,88)	13,95 (4,27)	10,55 (3,52)	4,20 (5,59)	33,65 (19,35)	1,15 (0,37)	30,05 (13,49)
Hyb	EFS17	49,46 (18,16)	0 (0)	12,14 (4,02)	13,62 (4,41)	10,62 (3,69)	1,15 (2,98)	17,54 (11,60)	0,85 (0,38)	31,85 (13,88)
Hyb	EFS18	64,47 (19,99)	0 (0)	19,73 (5,74)	21,74 (5,99)	17,58 (5,25)	0 (0)	19,21 (19,56)	0,68 (0,48)	16,32 (12,97)
Hyb	EFS19	44,13 (16,96)	0 (0)	20,81 (10,94)	22,96 (11,01)	19,00 (11,04)	6,01 (7,74)	16,87 (13,27)	0,87 (0,55)	32,99 (14,38)
Hyb	EFS21	52,30 (18,67)	0 (0)	19,15 (7,68)	21,26 (7,96)	17,09 (7,52)	0,12 (0,58)	13,96 (14,01)	0,61 (0,5)	33,62 (21,92)
Hyb	EFS22	24,44 (9,95)	0 (0)	10,93 (6,30)	12,83 (6,39)	9,28 (6,29)	13,67 (11,60)	30,50 (13,48)	1,28 (0,57)	31,38 (12,03)
Q. ilex	Ilex01	16,86 (7,01)	0 (0)	10,27 (3,33)	11,14 (3,53)	10,00 (3,16)	0 (0)	13,86 (11,02)	1 (0,82)	69,29 (15,35)
Q. ilex	Ilex02	21,40 (5,46)	0 (0)	10,95 (3,11)	11,90 (3,51)	10,10 (2,77)	0 (0)	8,90 (10,69)	1,1 (0,88)	69,70 (13,95)
Q. suber	Suber	99,08 (1,62)	1,00 (0)	100 (0)	100 (0)	100 (0)	0 (0)	0,92 (1,62)	0,33 (0,49)	0 (0)

Table S2: Pearson correlation estimate between the anatomical variables of the barks described in Table 1.

	PPH	PHT	PT	Nmax	Nmin	DS	PS	SG	PP
**PPH**	1								
**PHT**	0,452	1,000							
**PT**	0,626	0,812	1,000						
**Nmax**	0,631	0,793	0,994	1,000					
**Nmin**	0,615	0,825	0,996	0,984	1,000				
**DS**	-0,396	-0,123	-0,193	-0,198	-0,189	1,000			
**PS**	-0,540	-0,219	-0,345	-0,344	-0,339	0,074	1,000		
**SG**	-0,143	-0,095	-0,130	-0,131	-0,125	0,022	0,194	1,000	
**PP**	-0,583	-0,314	-0,385	-0,389	-0,379	-0,046	-0,264	-0,006	1,000
